# Perioperative aspirin and long-term survival in patients undergoing coronary artery bypass graft

**DOI:** 10.1038/s41598-018-35208-7

**Published:** 2018-11-19

**Authors:** Qian Ding, Hong Liu, Zugui Zhang, Jordan Goldhammer, Eric Yuen, Zhongmin Li, Linong Yao, Nilas Young, Douglas Boyd, William Weintraub, Rohinton Morris, Jianzhong Sun

**Affiliations:** 1 Department of Anesthesiology, Thomas Jefferson University, Philadelphia, PA 19107 USA; 2 Anesthesiology and Critical Care, Tangdu Hospital, The Fourth Military Medical University, Xian, 710038 P. R. China; 3 Department of Anesthesiology and Pain Medicine, University of California Davis Medical Center, Sacramento, CA 95817 USA; 4 Department of Section of Cardiology, Center for Outcomes Research, Christiana Care Health System, Newark, DE USA; 5 Sidney Kimmel Medical College, Thomas Jefferson University, Philadelphia, PA 19107 USA; 6 Department of Internal Medicine, University of California Davis Medical Center, Sacramento, CA 95817 USA; 7 Division of Cardiothoracic Surgery, University of California Davis Medical Center, Sacramento, CA 95817 USA; 8 Division of Cardiothoracic Surgery, Thomas Jefferson University, Philadelphia, PA 19107 USA

## Abstract

This study aimed to examine association between perioperative uses of aspirin and long-term survival in patients undergoing CABG. A retrospective cohort study was performed in 9,584 consecutive patients receiving cardiac surgery from three tertiary hospitals. Of all the patients, 4,132 patients undergoing CABG met inclusion criteria and were divided into four groups: with or without preoperative or postoperative aspirin respectively. 30-day postoperative and long-term mortality were compared with the use of propensity scores and inverse probability weighting adjustment to reduce the treatment-selection bias. The patients taking preoperative aspirin presented significantly more with comorbidities. However, the results of this study showed that preoperative aspirin (vs. no preoperative aspirin) was associated with significantly reduced the risk of 30-day mortality in the patients undergoing CABG. Further, the results of long-term mortality showed that the patients taking preoperative aspirin and postoperative aspirin (vs. not taking) were associated with significantly reduced the risk of 4-year mortality (14.8% vs. 18.1%, RR: 0.82, 95% CI: 0.75–0.89, P = 0.005; 10.7% vs. 16.2%, RR: 0.66, 95% CI: 0.50–0.82, P = 0.003). In conclusion, this cohort study showed that perioperative (before and after surgery) use of aspirin was associated with significant reduction in 30-day mortality without significant bleeding complications, also improved long-term survival in patients undergoing CABG.

## Introduction

Aspirin is one of the most common used drugs in preventing and treating cardiovascular disease (CVD) and its complications. In 2002, Antiplatelet Trialists’ Collaboration led by Baigent *et al*., a meta-analysis on about 287 trials (n = 212,000), has demonstrated that aspirin significantly reduced rates of myocardial infarction (MI), stroke and vascular mortality among the high-risk patients (secondary prevention) for long term use^1^. Concerning aspirin use in patients with CABG, however, the outcome results from randomized clinical trials (RCTs) and observational studies have been mixed and inconsistent^2–4^. Several meta-analyses in the area have showed that preoperative aspirin reduced perioperative MI, but was associated with an increased risk of reoperation, transfusion and bleeding. However, these meta-analyses were based upon early RCTs that were small and underpowered for efficacy outcomes^2–4^. The observational studies in the area showed that preoperative aspirin was associated with a decreased risk of perioperative complications and death, with or without an increased risk of bleeding^5–9^. In 2011, the American Heart Association and American College of Cardiology (AHA/ACC) recommended that aspirin (100 mg-325 mg daily) should be administered to CABG patients preoperatively^10^, although conflicting guidelines existed concerning preoperative aspirin and cardiac surgery^11,12^. In 2016, a large RCT led by Myles *et al*. showed that among 2,100 patients undergoing CABG with or without valve surgery, preoperative aspirin (100 mg) given on the day of surgery did not reduce risk of primary outcome including 30-day death, MI, stroke, renal failure, pulmonary embolism or bowel infarction, nor did increase the risk of bleeding^13^.

The length of follow-up is one of major limitations in most previous studies in the area of aspirin effects on cardiac surgery. Moreover, it remains unknown about perioperative aspirin’s effect on the long-term survival in patients undergoing CABG surgery. Thus far, a few studies have showed that aspirin started before or after surgery, improved vein graft patency up to 1 year after CABG^14–16^. Thus, this study aimed to examine association between perioperative (before and after surgery) use of aspirin and long-term survival in patients undergoing CABG.

## Results

### Characteristics of the study patients

**Figure 1:**
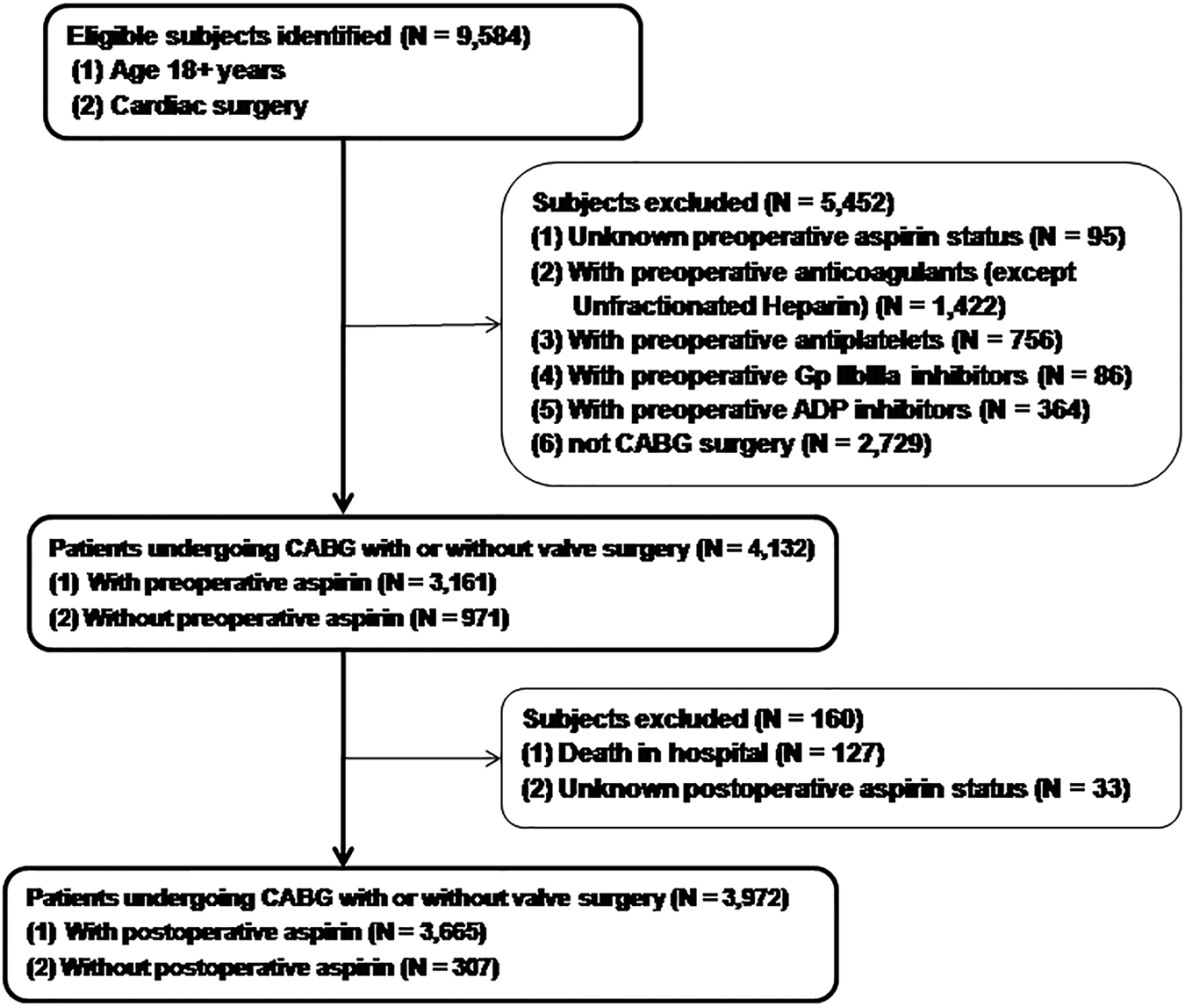
Study population recruitment summary. ADP, adenosine diphosphate; CABG, Coronary Artery Bypass Grafting.

**Figure 2:**
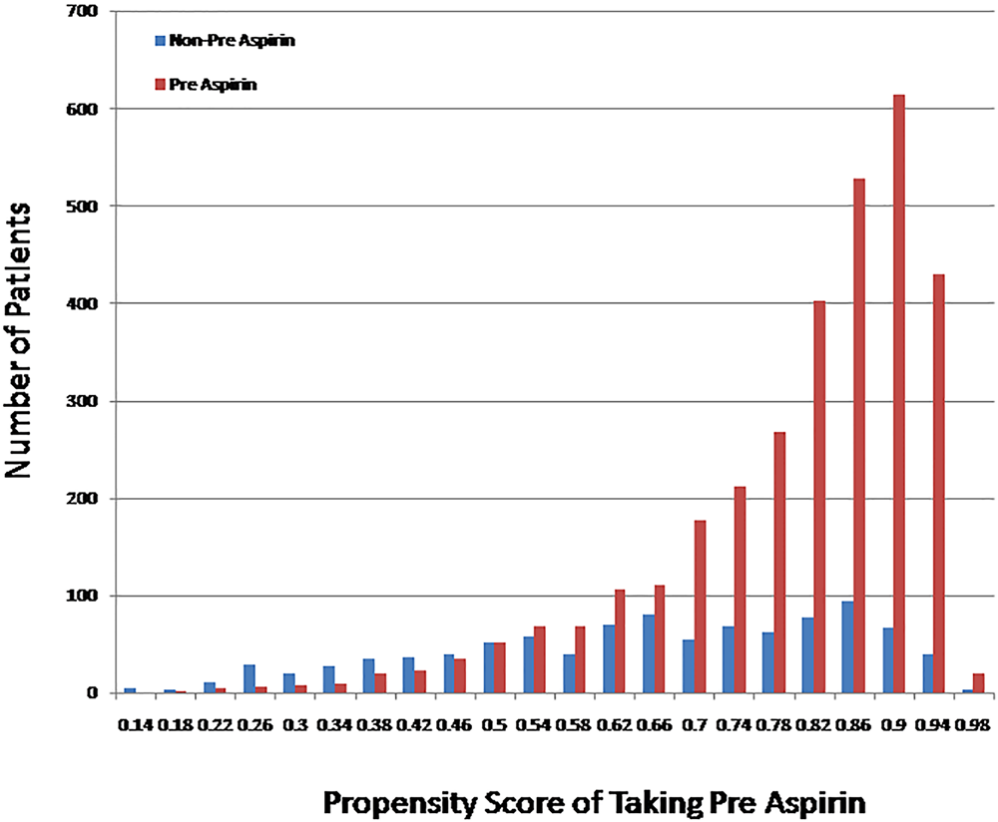
Propensity Scores for Preoperative Aspirin in Patients undergoing Coronary Artery Bypass Grafting (CABG) Surgery. The propensity score for Preoperative Aspirin is the probability given baseline variables that any patient in either group would take aspirin before cardiac surgery.

Among 4132 included patients, 76.5% received preoperative aspirin (PreASA), 23.5% did not (no-PreASA), 92.3% received postoperative aspirin (PostASA) and 7.7% did not (no-PostASA) respectively (Fig. 1). The patients’ demographic and clinical data are presented in Table 1. Before adjusted with using IPW, more patients taking aspirin than not taking one had smoking, diabetes, peripheral vascular disease, angina, hypertension, previous MI, required urgent surgery and underwent CABG, but less underwent CABG + valve surgery and took shorter time on bypass and cross-clamp; they were more with family history of CAD, more taking lipid lowering drugs, ACE inhibitors or beta blockers, but less with history of congestive heart failure, bypass time and cross clamp time. After adjustment with IPW, most of clinical covariates were well balanced and no significant differences were found between the two groups (Table 1). Figure 2 showed the propensity scores distribution between two groups, illustrating that patients taking preoperative aspirin had a higher probability of being selected for taking preoperative aspirin than those not taking aspirin. The mean, median and interquartile range of the propensity scores for preoperative aspirin reflected these differences (PreASA group: mean, 80.3%, median, 84.4%; interquartile range, 74.5% to 90.4%; No-PreASA group: mean, 64.0%, median, 67.1%; interquartile range, 49.4% to 74.5%).

**Table 1:** Demographic and Clinical Characteristics of Patients (PreASA).

Characteristic	Unadjusted Data		P Value	Adjusted Data (IPW)		P Value
	PreASA (N = 3161)	no-PreASA (N = 971)		PreASA (N = 3161)	no-PreASA (N = 971)	
Age, mean (SD), y	66.3 (10.8)	64.1 (13.6)	<0.001	66.0 (12.6)	66.8 (25.1)	0.072
Male sex, %	79.0	70.1	<0.001	49.1	49.7	0.595
BMI, mean (SD), kg/m^2^	29.1 (6.0)	28.8 (6.0)	0.143	29.1 (6.9)	29.3 (12.7)	0.188
Diabetes, %	39.0	29.4	<0.001	37.1	40.5	0.001
Smoker, %	42.1	38.1	0.026	41.7	44.1	0.031
Hypertension, %	84.2	70.7	<0.001	81.5	83.2	0.042
Cerebrovascular disease, %	15.3	15.0	0.816	15.4	17.4	0.016
Peripheral vascular disease, %	14.4	10.9	0.005	13.9	15.6	0.033
Chronic lung disease, %	20.5	18.9	0.252	20.1	20.8	0.438
Family history CAD, %	47.5	33.6	<0.001	44.2	46.4	0.046
Creatinine, mean (SD), mg/dl	1.2 (1.1)	1.3 (1.3)	0.132	1.3 (1.3)	1.3 (2.3)	0.826
Angina, %	46.6	30.3	<0.001	42.9	42.4	0.630
Congestive heart failure, %	22.3	32.5	<0.001	24.9	24.3	0.460
Previous MI, %	37.9	26.9	<0.001	35.3	35.9	0.570
Beta blockers, %	77.0	50.0	<0.001	70.7	71.5	0.427
ACE inhibitors or ARB, %	42.8	33.1	<0.001	41.1	42.2	0.311
Lipid lowering, %	65.3	44.4	<0.001	60.5	61.5	0.361
Urgent status, %	54.3	44.6	<0.001	51.9	49.8	0.061
Initial ICU Hours	92.4 (127.5)	104.9 (137.0)	0.01	94.5 (151.6)	99.2 (257.9)	0.240
CABG, %	83.8	71.3	<0.001	80.5	79.7	0.353
CABG and Valve, %	16.2	28.7	<0.001	19.5	20.3	0.353
Cardiopulmonary Bypass Time (SD), min	118.5 (73.8)	143.4 (92.6)	<0.001	124.6 (91.9)	123.7 (171.8)	0.781
Cross Clamp Time (SD), min	85.8 (55.2)	100.2 (66.4)	<0.001	89.2 (66.1)	89.0 (128.2)	0.934

BMI, body mass index, is the weight in kilograms divided by the square of the height in meters; MI, myocardial infarction; CAD, coronary artery disease; ACE, angiotensin-converting enzyme; ARB, Angiotensin Receptor Blockers; CABG, Coronary Artery Bypass Grafting; SD, standard deviation.

### Preoperative aspirin, 30-day and long-term mortality

Of 4132 patients included in this study, 3161 and 971 patients took or did not take preoperative aspirin, and showed 30-day mortality of 3.01% with PreASA and 4.74% without PreASA, respectively. We used multivariate analyses to assess independent risk factors for 30-day mortality, and found that preoperative aspirin was associated with the reduced incidence of mortality (OR 0.707, 95% CI 0.559–0.894, P < 0.01). As the value of C statistic showed, the multivariate regression analysis for preoperative aspirin, achieved an acceptable discrimination between two groups (C = 0.747; 95% CI: 0.729–0.765; P < 0.001).

**Figure 3:**
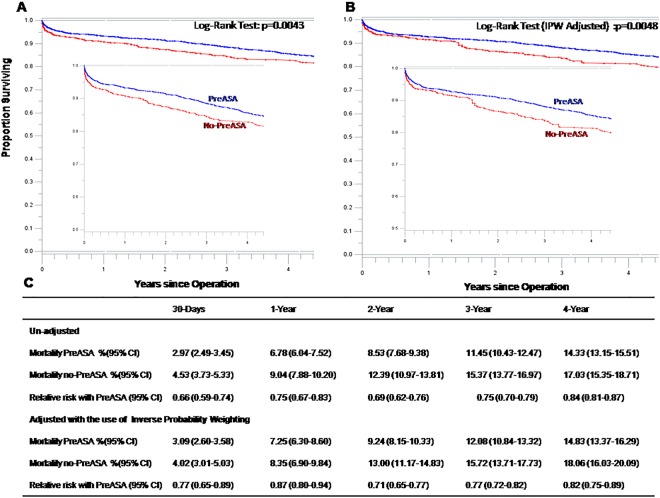
Rates of Survival in patients with preoperative aspirin (PreASA) or no-preoperative aspirin (no-PreASA). (**A**) From an un-adjusted Analysis. (**B**) From an analysis adjusted with the use of Inverse Probability Weighting (IPW). The inset shows the same data on an enlarged y axis. (**C**) Cumulative mortality with and without preoperative aspirin, and the relative risk of PreASA as compared with no-PreASA are shown.

The mean, median, and interquartile range of the follow-up time for 4132 patients were 4.49 years, 3.77 years, and 1.39 to 7.16 years, respectively. Figure 3A,B showed unadjusted and adjusted survival curves in patients taking or not taking preoperative aspirin respectively. At 1 year, a significant difference was found in adjusted mortality between the groups (7.25% in preoperative aspirin group vs. 8.35% without preoperative aspirin group; RR, 0.87; 95% CI, 0.80 to 0.94). The survival benefits were also seen from preoperative 2-year to 4-year mortality changes. The adjusted 4-year mortality was 14.83% in preoperative aspirin group vs. 18.06% without preoperative aspirin group (RR, 0.82; 95% CI, 0.75 to 0.89).

### Postoperative aspirin and long-term survival

**Figure 4:**
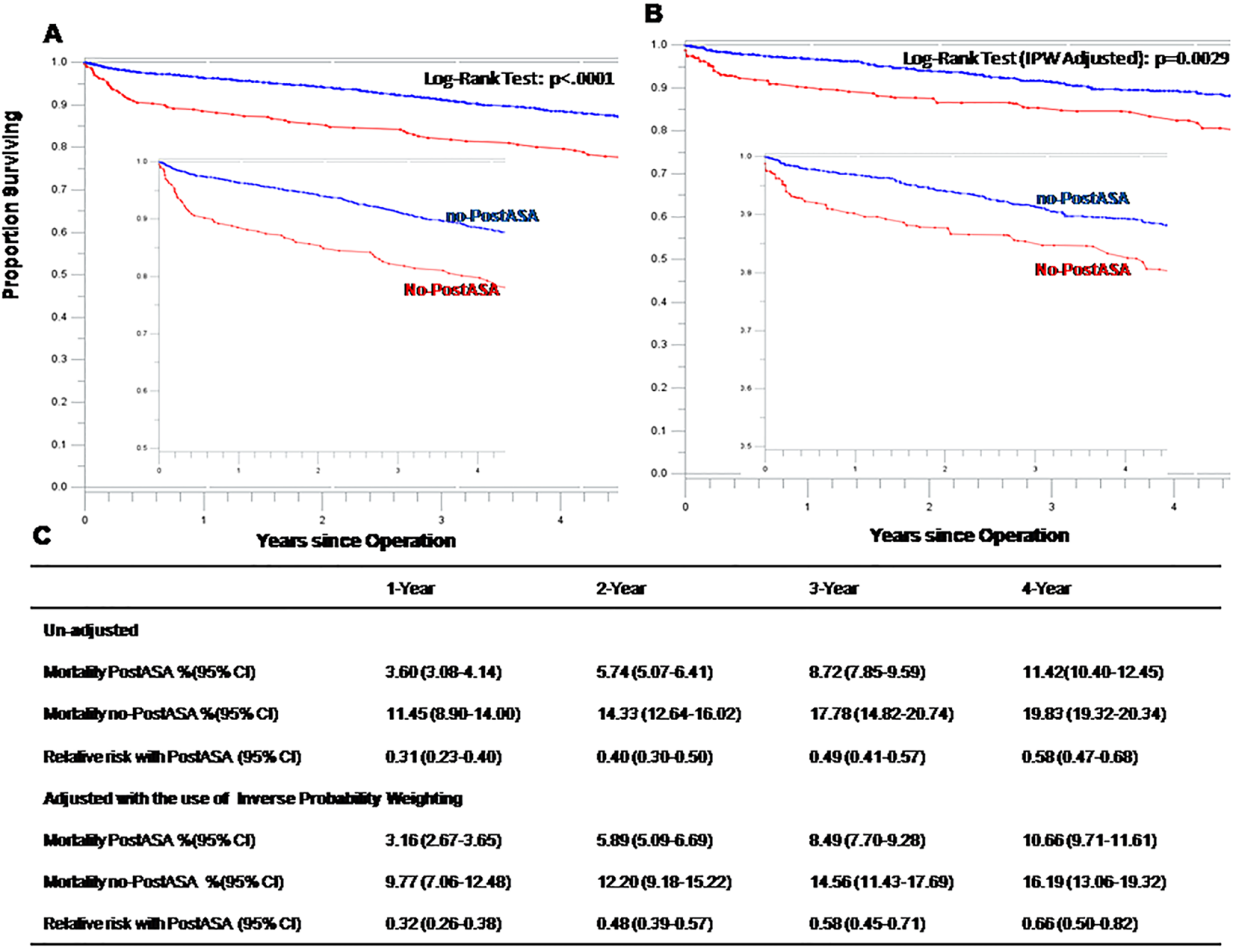
Rates of survival in patients with postoperative aspirin (PostASA) or no-postoperative aspirin (no-PostASA). (**A**) From an un-adjusted Analysis. (**B**) From an analysis adjusted with the use of Inverse Probability Weighting (IPW). The inset shows the same data on an enlarged y axis. (**C**) Cumulative mortality with and without postoperative aspirin, and the relative risk of PostASA as compared with no-PostASA are shown.

Based on discharge prescriptions after surgery, 3972 patients were divided into two groups: those prescribed (3665) or not prescribed (307) postoperative aspirin (Fig. 1). The follow-up time for those 3972 patients was the same as preoperative aspirin (4 years on average). Overall, use of postoperative aspirin is associated with significant beneficial effects on long-term survival. Figure 4A,B showed unadjusted and adjusted survival curves in patients prescribed or not prescribed postoperative aspirin respectively. At 1 year, a significant difference was found in adjusted mortality between the groups (3.16% in postoperative aspirin group vs. 9.77% without postoperative aspirin group; RR, 0.32; 95% CI, 0.26 to 0.38). This survival benefit continued throughout 4-years follow-up. The adjusted 4-year mortality was 10.66% in postoperative aspirin group vs. 16.19% without postoperative aspirin group (RR, 0.66; 95% CI, 0.50 to 0.82).

### The average adjusted hazard ratio and effect of unmeasured confounding

With the use of the Cox model analysis, the estimated average covariate-adjusted hazard ratio with preoperative aspirin as compared with non-preoperative aspirin was 0.801 (95% CI: 0.742–0.860, p-value = 0.017), slightly larger than the estimated 4-year risk ratio (0.78) with obtained with wider 95% CI (0.71–0.85) via the use of IPW approach. Similarly, for postoperative aspirin as compared with non-postoperative aspirin, the estimated average covariate-adjusted hazard ratio was 0.643 (95% CI: 0.58–0.71).

**Figure 5:**
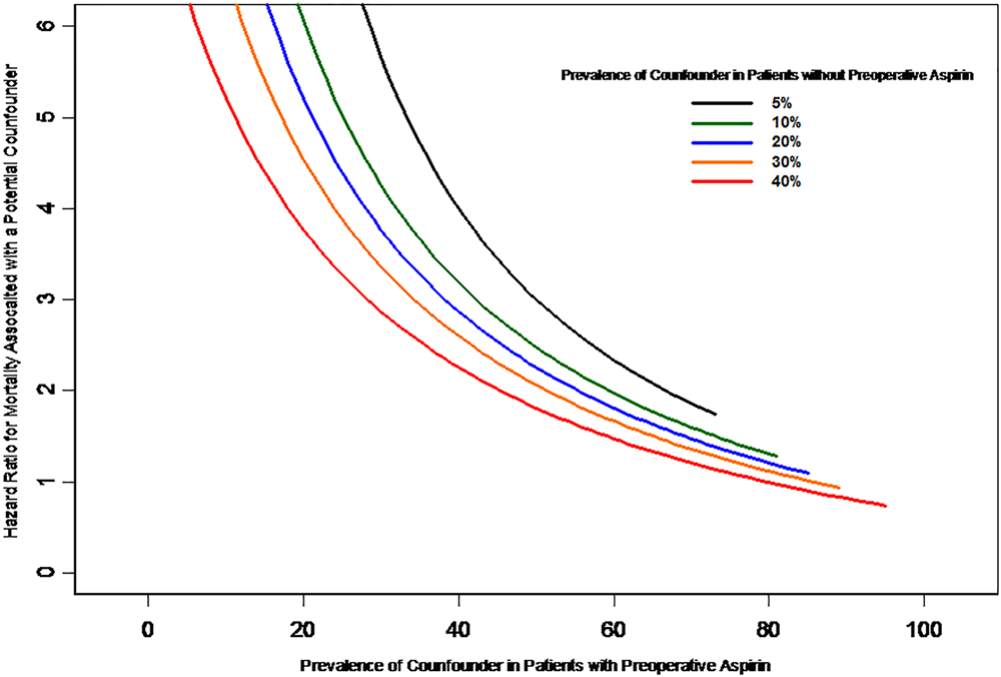
The average adjusted hazard ratio and effect of unmeasured confounding. The impact of unmeasured confounding factors on the hazard ratio has been one of the crucial uncertainties. Figure 5 shows the impact of a single confounder on the benefits of preoperative aspirin over without preoperative aspirin detected in the IPW adjusted analysis. If a single unmeasured confound could increase the long-term risk of mortality by a factor or about 1.7 or if the long-term mortality rate was two to six times as high among the preoperative aspirin patients as in the non-preoperative aspirin patients, it could generate the observed survival differences. As we can see that if a potential confounder was present in 40% of the non-preoperative aspirin patients (red curved line) and in 60% of pre-operative aspirin (X-axis), and if it increased the mortality rate by a factor of about 1.7 (hazard ratio, 1.81), the confounder alone could constitute the observed difference in mortality between preoperative aspirin and non-preoperative aspirin patients.

The impact of an unmeasured confounding is shown in Fig. 5. We used the X-axis to denote the assumed prevalence of the unmeasured confounder in the preoperative aspirin patients, and the Y-axis to denote the assumed hazard ratio for mortality related to this binary risk factor. We considered the assumed prevalence of the binary unmeasured confounder in non-preoperative at 5%, 10%, 20%, 30%, and 40%, respectively; and then we calculated corresponding hazard ratio related to the unmeasured confounder to explain the observed decreased risk. For instance, if an unmeasured binary risk factor was shown in 20% of the non-preoperative aspirin (blue curved line) and in 20%, 40%, or 60% of the preoperative aspirin, then using the method by Lin. *et al*.^17^ we obtained the hazard ratios for the unmeasured confounder to explain the observed decreased risk with non-preoperative aspirin would be 5.83, 4.17, and 2.08, respectively.

## Discussion

The results of this cohort study showed that preoperative and postoperative aspirin therapy was associated with a significant long-term survival benefit among the patients undergoing CABG. In addition, preoperative aspirin was associated with a reduced risk of postoperative 30-day mortality without increased bleeding complications^9^ in patients undergoing CABG. Overall, the findings reveal that benefits of perioperative uses of aspirin exceed hazards in patients undergoing CABG, in particular, perioperative uses of aspirin showed a strong association with improved long-term survival in the real world practice.

In the area of the research on aspirin and cardiac surgery, it is still uncertain and controversial about aspirin’s effects in patients with CABG, partially because it has been lacking of the hard-endpoint result–long term mortality. It was difficult to appreciate the overall picture of aspirin’s effects on cardiac surgery patients due to lacking of clinical evidence: long-term survival. Previous clinical studies have clearly showed that the treatment effect on chronic CVD takes a long time to emerge. For example, early clinical studies of vein grafts have shown that perioperative administration of aspirin improves the graft patency up to 12 month after the operation^14–16^. The SYNTAX^18^ and ASCERT^19^ trial demonstrated that the benefits of CABG vs PCI did not occur until 3–5 years later after the operation. The longitude of this study is extended from immediate postoperative time to an average of 4-years. The results of the present study revealed that the treatment effect with preoperative aspirin regarding the primary outcome – the long term survival (15–20% improvement) was similar to that observed in Antiplatelet Trialists’ Collaboration^1^ and also other proven medical treatments^20,21^, indicating that aspirin is an effective drug in improving long-term survival of patients with CABG.

With regard to aspirin’s short term effects, a recent RCT, ATACAS trial^13^ showed that among patients undergoing CABG with or without valve surgery or another procedure, the administration of preoperative aspirin resulted in neither a lower risk of death or thrombotic complications nor a higher risk of bleeding than that with placebo. The absence of an adverse bleeding effect in this trial is in line with previous clinical studies^5–9^ adding new evidence that a low-dose aspirin does not have significant effect on bleeding in patients undergoing cardiac surgery. The study should be commended for their strength of randomization. Nonetheless, the hard end-point - the event rate of death in this study was very low: there were only 14 vs 9 deaths in the treated (n = 1047) and placebo (n = 1053) groups, indicating a low-risk cohort of patients included in the study.

The beneficial effects of aspirin, in particular, its anti-inflammatory and/or pleiotropic effects most likely take time to occur. On this front, recent studies on potential mechanisms for aspirin in preventing atherothrombosis and chemoprevention on cancer have showed that, apart from suppressing thromboxane (TX) A2 production and TXA2-mediated platelet activation and aggregation, aspirin may play its multifaceted clinical effects via acetylating proteins in blood coagulation, inhibiting COX-2 activity and a variety of COX-independent mechanisms^22^. The underlying rationale for this study was that aspirin, for the long-term use, would prevent events related to its anti-inflammation and pleiotropic effects, in addition to those related to anti-platelet and anti-thrombosis.

The present study provided additional findings regarding effects of preoperative aspirin, including the association with reducing 30-day mortality without significant bleeding complications; the findings complement those of previous studies^5–9,23^. In addition, in a separate study^9^, we examined preoperative aspirin and bleeding complications in patients undergoing cardiac surgery. The results showed that preoperative aspirin did not significantly increase bleeding complications, including chest tube drainage, reoperation for bleeding. As it is known, CABG surgery frequently provokes an extreme and complex stress and hypercoagulable state, with an increased predisposition to long-term vascular morbidity and mortality. Perioperative aspirin may provide cardiovascular protection with potential short- and long-term benefits for survival.

### Limitations of this study

First, we have applied the propensity scores, IPW and other methods in this study to minimize biases and control confounding, however, the potential flaws of a non-randomized study may still exist. Second, other unknown confounding factors may exist which could affect the outcomes, thus we did an analysis as described in Fig. 5. With regard to longitudinal data and/or survival analysis, we selected the method of IPW due to its advantages in the analysis in comparison with the methods of matching and stratification^24,25^. Third, clinically it would be appropriate to group the patients based on whether they were taking aspirin during both the pre- and post-operative period (continuing aspirin) compared with patients they were not on aspirin at any point during the perioperative period (no aspirin). However, a timespan of the cohort follow-up between preoperative aspirin and postoperative aspirin prescription is “immortal” since exposed patients who received their first prescription (preoperative aspirin) had to survive until their second prescription (postoperative aspirin), which introduces an immortal time. Thus, classifying aspirin into 2 groups (continuation vs. none) has a problem due to the difference exposure (immortal time bias) of the groups^26^. Fourth, we lacked data on the dose and adherence of aspirin in this cohort of patients (due to lack of refill records and aspirin being an over-the-counter drug). However, the discharge prescription of aspirin often is indicated for patients with CABG and it should be continued indefinitely and the usual doses are 81 mg–325 mg daily, except for ones with contraindications. In addition, the reported rates of patient aspirin adherence for cardiovascular protection are high, range from 72% to 92% in the literature^27^. The association we found in this study suggests that perioperative aspirin may provide cardiovascular protection with potential long-term benefits for survival. The results of this study also indicate that further studies, including basic mechanistic studies and large clinical studies, both RCTs and pragmatic studies are still needed to assess aspirin effectiveness in patients undergoing cardiac surgery.

## Conclusions

Among patients undergoing CABG, taking aspirin before and after surgery was associated with a significant reduced mortality risk during 4-years of follow-up.

## Methods

### Study design

A retrospective and cohort study was performed in patients (n = 9584) undergoing cardiac surgery at three teaching hospitals (Thomas Jefferson University hospital, Abington Memorial hospital and UC Davis Medical Center, dated from 2001 to 2015). The study was in accordance with the Declaration of Helsinki^28^, approved by the local Institutional Review Board (IRB) including Thomas Jefferson University IRB and UC Davis IRB, and individual consent was waived in compliance with the HIPAA regulations and the waiver criteria. Preoperative and postoperative uses of aspirin are defined as within 5 days preceding surgery and on the discharge respectively. Of all patients, 4,132 patients undergoing CABG with or without valve surgery met the inclusion criteria and were divided into four groups: with or without preoperative (within 5 days preceding surgery) or postoperative (on discharge) aspirin respectively (Fig. 1).

### Data collection

The following data were collected: patient demographics, history, medical record information, preoperative risk factors, preoperative medications, intraoperative data, postoperative 30-day all-cause mortality, discharge medicines and long-term all-cause mortality. For missing at completely random and low missing rate variables, missing data values will be imputed using multiple multivariate imputation by chained equations for continuous variables (to enhance prediction of the missing value, relevant variables were stratified), and the most frequent value for categorical variables. For missing at random, each missing value was replaced with multiple imputation procedure, which used a set of plausible values to impute^19,29^.

### Measurement of Outcomes

Primary endpoints were 30-day and long-term mortality, which were based on the data Registry of our three hospitals and the Social Security Death Index^30^. The survival time (time-to-event) of the subject began when the subject had CABG, and ended when the end-point (the death) was reached or the subject was censored from the study^31^.

### Adjustment for differences between groups

As anticipated, patients with or without preoperative or postoperative aspirin would differ significantly regarding baseline (before surgery) characteristics. Using multilevel logistic regression, a propensity score was derived on the patient characteristics, reflecting the probability that a patient would receive preoperative aspirin. The individual variables included in the propensity model are listed in Table 1. The inverse probability weighting (IPW) approach based on the propensity scores was then applied as the tool to adjust for differences between the groups^32^. The performance of propensity model was verified by comparing the distribution of covariates and propensity scores between groups both before and after the IPW^33^.

### Statistical analysis

The Pearson chi-square test and Wilcoxon rank-sum test were used for analyzing continuous or categorical variables respectively. For long-term survival, survival curves were estimated with using the Kaplan-Meier method (unadjusted)^34^, then re-estimated with using the IPW approach (adjusted) by Cole and Hernan^35^. For each group with or without preoperative or postoperative aspirin, the survival curves adjusted with the use of IPW represent the expected rate of survival if the treatment of interest were applied to all study patients. Risk ratios at specific time points were calculated with estimated rates of survival among patients receiving CABG with or without preoperative and postoperative aspirin, 95% confidence intervals were obtained with bootstrap methods.

To conduct sensitivity analysis, survival curves were re-estimated separately for patients with or without preoperative and postoperative aspirin with the use of Cox proportional-hazard models without propensity scores^36^. The same covariates were used in each model as those used in the propensity model above. Further, to examine the impact of potential unmeasured confounders, we conducted covariate-adjusted Cox modelling to estimate average hazard rations for preoperative aspirin group vs. non-preoperative aspirin, as well as post-operative aspirin vs. non-aspirin. Additionally, we evaluated if the observed differences in the mortality rate could be thoroughly explained via an unmeasured confounder on the basis of the method outlined by Lin *et al*.^17^. Percentages, relative risk (RR), hazard ratio (HR), 95% confidence intervals (CI) and P values (2-sided) <0.05 were given in the results. SAS version 9.4 (SAS Institute, Inc, Cary, NC) and SPSS 17.0 software for Windows (SPSS Inc., Chicago, IL) were used for the statistical analysis.

## Data Availability

All data generated or analyzed in this study are available from the corresponding author on reasonable request.
